# Caring for Those Who Care

**DOI:** 10.3390/bs15070883

**Published:** 2025-06-28

**Authors:** Eduardo Fernández-Jiménez, Clara González-Sanguino

**Affiliations:** 1 Department of Psychiatry, Clinical Psychology and Mental Health, La Paz University Hospital, 28046 Madrid, Spain; 2 Hospital La Paz Institute for Health Research (IdiPAZ), 28046 Madrid, Spain; 3 Faculty of Law, Education and Humanities, Universidad Europea de Madrid, 28670 Villaviciosa de Odón, Spain; 4 Department of Psychology, School of Education and Social Work, University of Valladolid, 47011 Valladolid, Spain; clara.gonzalez.sanguino@uva.es

## 1. Introduction

Healthcare professionals are exposed to numerous adverse stressors and very demanding settings and, consequently, the levels of depression, anxiety, and stress-related disorders in this population are highly relevant, especially during the COVID-19 pandemic and in the post-pandemic context. They are, however, dependent on the measurement instruments used, the geographical region, and time. Interestingly, the prevalence of depression among healthcare workers before the COVID-19 pandemic was generally estimated to be less than 10% (Winkler et al., 2021); however, during this global health emergency, estimates of the prevalence of depression among healthcare workers increased noticeably, with pooled estimates ranging from approximately 22% (Li et al., 2021) to 36% (Sun et al., 2021). Additionally, estimates for anxiety disorders during the COVID-19 pandemic ranged from 22% (Li et al., 2021) to 40% (Saragih et al., 2021). Finally, the prevalence of stress-related disorders (post-traumatic stress disorder and acute stress) during this global health crisis was reported to range from 21.5% (Li et al., 2021) to 49% (Saragih et al., 2021).

In this context, burnout levels are a worrying problem among healthcare workers, both prior to and following the COVID-19 pandemic. Specifically, the prevalence of burnout before the pandemic was approximately 30–31% among USA healthcare workers, with rates remaining stable from 2018 to 2020. In contrast, during the pandemic, the prevalence of burnout increased significantly, reaching a peak of 39.8% in 2022 before declining to 35.4% by 2023. However, these levels remained elevated compared with those observed before the pandemic (Mohr et al., 2025). Moreover, international meta-analyses conducted during the pandemic have indicated even higher rates, with the global prevalence of burnout among healthcare workers rising to approximately 55%. In addition, the highest prevalence rates of burnout (approximately 66%) have been observed in countries in the Middle East and North Africa (Macaron et al., 2023).

Owing to its multifaceted nature (which includes emotional exhaustion, depersonalization/cynicism, and reduced personal accomplishment), burnout is a complex construct that requires a nuanced approach. Compassion fatigue, another construct that is closely linked to burnout, is defined as an impaired ability to care as a consequence of repeated exposure to patient suffering (“vicarious trauma”) (Cavanagh et al., 2020). Both phenomena share the dimensions of emotional exhaustion and reduced personal accomplishment, although compassion fatigue is more specifically linked to empathic engagement with traumatized patients, and burnout is more broadly related to structural and organizational stressors (Cavanagh et al., 2020).

Nevertheless, healthcare workers do not experience psychological impacts uniformly, necessitating an intersectional approach to addressing these differences. Notably, nurses are the healthcare professionals most consistently and significantly affected by burnout, anxiety, and depression, particularly during the COVID-19 pandemic (Moll et al., 2022), and this pattern has been identified across diverse geographic regions and healthcare systems. Likewise, other professional roles (social workers, service workers, and administrative staff) also showed an increased risk of depression compared to physicians (Pala et al., 2022). Moreover, frontline workers (Tian et al., 2023) and those who maintained direct patient contact (especially in intensive care units; ICUs) (Azoulay et al., 2020) were the professional subgroups that were particularly vulnerable during the COVID-19 pandemic, with burnout prevalence increasing from 59% in 2017 to 69% in 2020 among ICU clinicians (Moll et al., 2022). In addition, female gender is another key factor to consider in this feminized population. In particular, during the COVID-19 pandemic, female healthcare workers reported more mental health problems compared to men, as well as less support from their colleagues (Czepiel et al., 2024). Moreover, a higher prevalence of burnout has been observed among female physicians than among their male counterparts (McClafferty et al., 2022). These phenomena probably owe much to the patriarchal culture that extends into the workplace—where women took on a greater burden of care at home during the pandemic and balanced this with their work—a state of affairs that, in the case of healthcare professionals, seems to continue to show marked gender differences.

Interventions devoted to healthcare workers must act at both the individual and organizational levels. In particular, at the individual level, effective interventions for reducing stress and depression symptoms include cognitive–behavioral therapy-based interventions (covering components of psychoeducation, emotion regulation, and problem-solving) and resilience training programs (Kunzler et al., 2020). In addition, other individual-level interventions that are effective at reducing stress and burnout levels are mindfulness-based programs (Catapano et al., 2023), which are successful regardless of the treatment delivery modality (in-person, online, therapist-assisted, or self-guided) and are especially feasible and useful for large-scale implementation in high-risk groups during a health crisis (Rodriguez-Vega et al., 2020). On the other hand, at the organizational level, the evidence is less robust than it is for individual-level interventions, such that there are as yet no recommended interventions effective at this level to reduce the levels of burnout for this specific population (Catapano et al., 2023).

In addition, institutions should design and implement educational interventions to reduce stigma towards mental health problems among healthcare professionals and trainees (Wong et al., 2024). A workplace free of stigma means that employees feel safe and respected, regardless of their circumstances and health issues. This not only improves their health and reduces burnout levels (Fannin et al., 2023; Favre et al., 2023) but, in the healthcare context, could also lead to better care for users, resulting in a win–win situation for everyone (Del Olmo-Romero et al., 2025). The facilitators for making all these interventions feasible are as follows: adaptation to the local needs of each specific healthcare center/department; fostering effective communication channels, both formally and informally; and promoting safe, positive, and supportive learning environments for frontline workers (Pollock et al., 2020).

## 2. Research by Health Outcomes

In light of the aforementioned considerations, this Special Issue covered a wide range of study designs aimed at examining the emotional and affective impacts—specifically, stress-related disorders, anxiety, and depression—associated with professional activity among healthcare workers. In this sense, this Special Issue mainly focused on a key syndrome for healthcare workers, such as burnout, and seven out of the ten studies included in this Special Issue addressed burnout as a relevant condition in this vulnerable population. One study investigated how different personality types, based on the Big Five model, were associated with burnout levels among Chinese nurses, indicating that nurses with a distressed personality profile were significantly more likely to experience burnout than those with a resilient personality profile (Contribution 1). Another study, by Surawattanasakul et al. (Contribution 2), studied the relationship between quality of work life (QWL) and burnout dimensions among first-year intern physicians in public hospitals in Thailand, showing that high levels of depersonalization and low levels of personal accomplishment were significantly associated with lower overall QWL. In addition, Yikilmaz et al. (Contribution 4) examined the negative impact of surface acting (faking emotions) on healthcare professionals, which increased job stress and emotional exhaustion (a burnout dimension), and found that strong leader–member exchange (i.e., trust and support from leaders) can contribute to reducing these negative effects. Similarly, Contribution 5 examined the factors influencing on the job burnout in the Jordanian public health workforce and analyzed the relationships between ethical leadership, organizational climate, and role overload, highlighting the need for ethical leaders and supportive work environments to reduce burnout, particularly when employees face heavy workloads. Moreover, Sikaras et al. (Contribution 6) showed that Greek nurses still experienced high levels of burnout and insomnia even after the end of the COVID-19 pandemic, and identified resilience as a protective factor that can help nurses cope with burnout and improve their sleep quality. Zamorano et al. (Contribution 8) studied the association between a stigma towards mental health problems and burnout levels among Spanish community social healthcare professionals, suggesting that addressing negative attitudes towards mental health and enhancing professional competencies could be crucial in terms of improving the well-being of these workers. Finally, Turunç et al. (Contribution 10) examined the relationship between burnout and turnover intention among intensive care unit (ICU) nurses in Turkey, identifying an association between increased burnout levels and a higher likelihood of leaving their jobs, and, unexpectedly, did not find that psychological resilience plays a significant mediating role in this relationship.

On the other hand, this Special Issue also encompassed studies that addressed predictors of other relevant outcomes for healthcare workers, such as self-efficacy, intention to leave the job, and personal impacts on mental health. Specifically, Contribution 3 was carried out during the COVID-19 pandemic and focused on how workplace frustration, a fear of COVID-19 infection, and working hours influenced healthcare professionals’ decisions to leave their jobs. Also, Milojević et al. (Contribution 9) analyzed how leadership styles impact the stress and self-efficacy of Serbian healthcare professionals and found that laissez-faire leadership approaches increased stress, whereas transactional leadership fostered self-efficacy levels. Finally, Stacchini et al. (Contribution 7) addressed the relationship between social support and depression among Italian medical residents in the public healthcare system and found that strong peer-to-peer and supervisor support, active social participation, and having a partner were associated with a lower risk of experiencing clinically relevant depressive symptoms. Interestingly, this Special Issue integrated international studies conducted in a broad range of countries, including China, Thailand, Turkey, Jordan, Greece, Serbia, Italy, Spain, and the United Kingdom.

## 3. Conclusions

This Special Issue included a comprehensive collection of studies that addressed burnout and stress-related problems among healthcare professionals, particularly focusing on their causes/predictors, consequences, and any potential mitigating factors.

Given the complex and specialized psychological care required by healthcare personnel, the integration of clinical psychologists into the interdisciplinary teams of Consultation-liason mental health units is essential. In this sense, clinical psychologists play a crucial role in supporting other healthcare professionals and in providing group supervision for teams in hospitals and other healthcare facilities. Therefore, the role of specialists in clinical psychology is key to promoting psychologically informed care environments in healthcare systems (Ebrahim, 2022). It is about caring for those who care.
